# Attempted suicide in Podgorica, Montenegro: higher rates in females and unemployed males

**DOI:** 10.1186/s12991-017-0151-5

**Published:** 2017-07-04

**Authors:** Lidija Injac Stevovic, Sanja Vodopic

**Affiliations:** 1 Clinical Department of Psychiatry, Clinical Centre of Montenegro, Podgorica, Montenegro; 2 Department of Psychiatry, School of Medicine, University of Montenegro, Dzona Dzeksona bb, Podgorica, Montenegro; 3 Clinical Department of Neurology, Clinical Centre of Montenegro, Dzona Dzeksona bb, Podgorica, Montenegro

**Keywords:** Attempted suicide, Montenegro, Rate, Risk factors, Unemployment

## Abstract

**Background:**

A change in suicide attempts is associated with comprehensive changes in mental and physical health and social environment. Attempted suicide and suicide are one of the biggest problems nowadays worldwide, not only in the field of mental health but also in the field of public health. The aim of the research was to determine the number of attempted suicides as well as the influence of clinical and demographic variables on the attempted suicide rate.

**Methods:**

The data on the attempted suicide were analysed in the period 2012–2016 based on the data from the Emergency Ward of the Clinical Centre of Montenegro in Podgorica. The rate of attempted suicides as well as the unemployment rate was calculated. The statistical analysis included descriptive statistics of the raw data and relative numbers, Chi-squared test, Fisher’s test and Spearman coefficient.

**Results:**

The average age of males who attempted suicide was 38.35 ± 14.11, min 15 and max 88 years of age, and the age of women was 38.97 ± 16.81, min 16 and max 93 years of age. Women attempted suicide more frequently (*p* < 0.05). Female/male ratio during the investigation period slightly declined (1.93 in 2012 vs. 1.29 in 2016). The attempted suicide rates ranged from 103 per 100,000 residents in 2016 to 142 per 100,000 residents in 2015. Crude attempt rate was the highest in women in 2012 (102.42 per 100,000 residents) and for men in 2014 and 2015 (84.48 vs. 83.06 per 100,000 residents). Poisoning with psychotropic drugs was the dominant manner of attempt (93.2%), while the largest number of attempts was in the late spring and summer (May, June and July). Attempted suicide rate in man was associated with higher unemployment rate.

**Conclusions:**

Although women make the majority of attempted suicide cases, there has been a decline in the value of the rate for women and a rise for men. The attempted suicide rates in Podgorica belong to lower rates compared to the WHO European multicentre study on parasuicide. Poisoning with psychotropic drugs was the predominant manner, while the highest number of attempted suicides was in the late spring and summer (May, June and July). Unemployment influences men to attempt suicide much more frequently.

## Background

Attempted suicide and suicide are one of the biggest problems nowadays worldwide, not only in the field of mental health but also in the field of public health. Little is known about attempted suicides in the world. It is estimated that in the countries of the WHO European Region, 150,000 people manage to commit suicide and 1,500,000 people attempt suicide annually [1].

According to the WHO data, the suicide rate in Montenegro in 2012 was 18.9/100,000 residents, which classified it in the tenth position in Europe [2].

Until 2009, Montenegro had official data on suicides which were published by the Statistical Office of Montenegro (MONSTAT) [3], while in 2010 this responsibility was assumed by the Institute of Public Health, and since then there have been no official data on suicides or attempted suicides. Podgorica is the capital of Montenegro. The latest statistical data show that the population of Podgorica is 187,075, which represents 30% of the total population of Montenegro. Men make 49.39% and women 50.61% of the population [3]. A research of attempted suicide is extremely important, because we are a small country, so that this “disadvantage” of ours could be an advantage in monitoring and faster identification of such individuals.

Attempted suicide is the strongest predictor of suicide or parasuicide [4], so that those who attempted suicide are an important group for monitoring. Attempted suicides are more frequent than commited suicides and very often lead to hospitalization. The most important risk factors identified are younger age and female gender, single or divorced, unemployed, recent change in living situation, mental disorder and previous parasuicide incident [5].

Despite the current knowledge, it remains difficult to reliably establish the risk of attempted suicide among individuals and in the community. That is why the studies on attempted suicides list many challenges arising from the very nature of suicide. The aim of the research was to determine the number of attempted suicides as well as the influence of clinical and demographic variables on the attempted suicide rate.

## Methods

The research was conducted in the Emergency Ward (EW) of the Clinical Centre of Montenegro (CCM) in Podgorica. We analysed the data on the attempted suicide in the period 2012–2016. The rate of attempted suicides and the unemployment rate were calculated. The data on population and the unemployment rate that we used were taken from the web site of the national Statistics Office MONSTAT [3]. CCM is the only hospital in Podgorica. The Emergency Ward operates 24 h a day within CCM. Each attempted suicide is registered on the basis of reports of patients or their families, as well as on the basis of the examinations carried out by psychiatrists and other specialists, including all relevant clinical and laboratory tests. All patients were accompanied by family members who provided additional information. All patients 18 years old and above, who attempted suicide in the period between 2012 and 2016, were included in the research. There were examined 608 patients in the Emergency Centre. The methodology will be used as a basis for the discussion on the situation with the population of Podgorica.

## Statistics

The analysis included descriptive statistics of the raw data (absolute numbers) and relative numbers. Mortality rates were standardized to the 2011 Montenegro population census using the direct method. Rates are per 100,000 individuals per year. For the distribution of the method, Chi-squared test and Fisher’s test were used. For the correlations of the unemployment rate and the attempted suicide, Spearman coefficient was used. The significance level was set at *p* < 0.05. Data analyses were performed in SPSS 16.0.

## Results

In the period 2012–2016, 608 respondents attempted suicide. The largest number of attempted suicides was in 2015—142 attempts (23.40% of the sample) and the lowest number was in 2016—103 attempts (16.90%). Female/male ratio during the period of monitoring was on a slight decline (2012—1.93, and 2016—1.29) (Table 1). The average age of males who attempted suicide was 38.35 ± 14.11, and the average age of women was 38.97 ± 16.81.

**Table 1:** Numbers of attempts concerning each sex in each year and the respective female-to-male ratio for absolute raw numbers

Year	Male		Female		Total	Female/male ratio
	Count	%	Count	%	Count	
2012	41	34.20	79	65.80	120	1.93
2013	41	37.60	68	62.40	109	1.66
2014	63	47.00	71	53.00	134	1.13
2015	69	48.60	73	51.40	142	1.06
2016	45	43.70	58	56.30	103	1.29

Attempted suicides were mostly performed using psychotropic drugs in the total population (93.2%). This method of attempting suicide is statistically much more frequent in women than men (95.10% vs. 90.50%, *p* = 0.043). Hanging and drowning are statistically more frequent in men (2.90% vs. 0.30%, *p* = 0.010) (Table 2).

**Table 2:** Distribution of method of attempting suicide by gender

	Total		Male		Female		*p* value^†^
	*N*	%	*N*	%	*N*	%	
Medication	551	93.20	220	90.50	331	95.10	0.043
Hanging/drawing	8	1.40	7	2.90	1	0.30	0.010^‡^
Toxic	11	1.90	6	2.50	5	1.40	0.545
Blunt object	20	3.40	9	3.70	11	3.20	0.898
Firearms	1	0.20	1	0.4	0	0	0.410^‡^

^†^Male vs. female, Chi-squared test, ^‡^ Fisher’s test

**Fig. 1:**
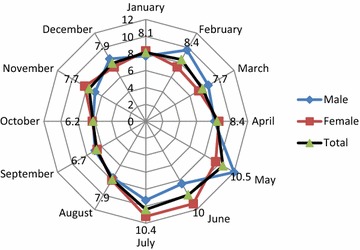
Seasonal character of attempted suicides

The highest frequency of attempted suicides was in May, June and July (10.50, 10.00 and 10.40%, respectively), i.e. during late spring and summer (Fig. 1). As for male patients, the highest frequency of attempted suicides was in May (12.00%), and when it comes to female respondents, the highest frequency was in June and July (11.20%).

Crude attempt rate was the highest in women in 2012—102.42 per 100,000 residents. As for male respondents, the peak of this rate was in 2014 and 2015 (84.48 and 83.06, respectively, per 100,000 residents) (Table 3).

**Table 3:** Attempt rates (per 100,000 residents) for the period 2012–2016 (standard population: Montenegro census 2011)

	Year	Regional population (15+)	Number of attempts	Attempt rate (per 100,000 residents)	Age-standardized attempt rate	95% CI for standardized attempt rate
Male	2012	73,297	41	55.94	58.99	40.93–77.05
	2013	73,779	41	55.57	63.38	43.98–82.78
	2014	74,571	63	84.48	68.09	51.28–84.90
	2015	75,851	69	83.06	73.13	55.87–90.39
	2016	76,782	45	58.61	78.60	55.63–101.57
Female	2012	77,131	79	102.42	99.46	77.53–121.39
	2013	77,639	68	87.58	93.15	71.01–115.29
	2014	78,472	71	90.48	87.24	66.95–107.53
	2015	79,818	73	91.46	81.70	62.96–100.44
	2016	80,798	58	71.78	76.52	56.83–96.21
Total	2012	150,428	120	79.77	63.96	52.52–75.40
	2013	151,417	109	71.97	68.44	55.59–81.29
	2014	153,042	134	87.56	73.23	60.83–85.63
	2015	155,669	142	91.22	78.36	65.47–91.25
	2016	157,580	103	65.36	83.85	67.66–100.04

Looking at the age-standardized attempt rate, we observe that during the monitoring period there is a balanced increase of the value of this rate in men, while there is a gradual decline of the value of age-standardized attempt rate among women for the same period. Age-standardized attempt rate for the whole population of the city of Podgorica follows the growth trend for the male population.

The highest correlation of the unemployment rate and the attempted suicide rate was observed in men (*ρ* = −0.800, and in the total population *ρ* = −0.600) (Table 4). While there is a negative correlation in men in the total population, there is a positive correlation (*ρ* = 0.205) in women between these two indicators. The small number of observations makes any use of statistical significance problematic.

**Table 4:** Correlation of the unemployment rate and the attempted suicides

Indicator	Attempted suicide rate		
	Males	Females	Total
Unemployment rate			
*Ρ*	−0.800	0.205	−0.600
*p*-value	0.145	0.741	0.285

*p*—Spearman coefficient, *p*-value < 0.05

## Discussion

This is the first study that has investigated attempted suicides in Podgorica, the capital of Montenegro. The paper deals with all the attempted suicides in the period of 5 years. During the observed period, 608 respondents attempted suicide, whereby the highest number was in 2015 and the lowest in 2016. The results show that the age of 38 in both sexes is the age of the highest risk for a suicide attempt. It has been observed that women attempted suicide more often than men, but in the monitoring period there was a slight decline of the attempted suicide in women compared to men (1.93/1.29). We could find similar data in similar Mediterranean countries such as Greece where the ratio is 2:1, which is a probable value for attempts, and 1:3.6 (more males) for committed suicides [6].

The largest number of attempts included poisoning with psychotropic drugs and statistically it occurs much more frequently in women, while, statistically, men rather use more lethal methods. These devastating results are in accordance with the studies conducted in England, Switzerland and Greece: over 75–95.93% attempted suicides were due to self-poisoning [6, 7]. It is worrying that overdosing is the most popular manner of attempt, while the others were used less frequently. It is a known fact that the availability of means leads to an increase of suicides. Control and distribution of psychoactive drugs in our country started only in mid-2016, which explains a lower rate of attempted suicides that year. Attempted suicides in men are more often linked with a strong suicidal intention and their attempts tend to be more lethal compared to women and more frequently point to a non-diagnosed mental disorder [8].

An epidemiological study in four European countries—Germany, Hungary, Ireland and Portugal—demonstrated that suicidal acts (fatal and non-fatal) were 3.4 times more lethal in men than in women and the proportion of serious suicide attempts among all non-fatal suicidal acts with known intentionality was significantly higher in men than in women [9]. In USA, males 1.6 times more likely than females use violent methods to attempt suicide [10].

The highest number of suicides took place during spring and summer. We identified three peaks: May, June and July. The increase in the number of suicides in May could be linked with the First May and the Independence Day holidays when alcohol is consumed and when man feel disappointed because of unemployment or return to work. June and July are the months when women have more expectations from holidays, which is followed by disappointment and dissatisfaction. Additionally, in Podgorica over the summer temperature is very high, about 40°, what has strong influence on the social life of the citizens.

In Greece, two peaks were identified for attempts—May and August (the border of summer) [6].

In Hungary, Seregi et al. [11] examined several environmental factors with periodic changes in intensity during the calendar year in order to explain the increase in suicide frequency during spring and summer. Their results strongly support the hypothesis that sunshine has a prompt, but very weak increasing effect on the risk of suicide (especially violent cases among males). The need to study the role of environmental factors in evoking suicidal behaviour is substantiated by the observation that suicide cases are unevenly distributed during the calendar year [12].

The attempted suicide rate in Podgorica ranged from 103 per 100,00 residents in 2016 to 142 per 100,000 residents in 2015, belonging to the group of the lowest rate compared to the WHO European multicentre study on parasuicide, where the male parasuicide rate varied from 45 to 314 per 100,000 and female parasuicide rate ranged from 69 to 462 per 100,000 of the population from the lowest to the highest level among the 13 participating countries [7].

As for age, standardized rates of attempts showed an increase in the value of rate for men, while for women there was a decline of the value, which shows that in Podgorica attempted suicide is not the behaviour that is characteristic not only for women but also for men. In Europe, the highest average male age-standardized rate of suicide attempts was found for Helsinki, Finland (314/100,000), and the lowest rate (45/100,000) was for Guipuzcoa, Spain. The highest average female age-standardized rate was found for Cergy-Pontoise, France (462/100,000), and the lowest (69/100,000) again for Guipuzcoa, Spain [7].

In Belgium, the rates are around 60–70 for males and 70–140 for females per 100,000 residents [13], and the same rates were recorded in Germany [14] and Sweden [14].

An increase of the rate of attempted suicides in men can be explained by the results of our research which point to a link of the unemployment rate and the attempted suicide rate, which means that unemployment represents a risk factor in men. Unemployment and low socioeconomic status represent important variables in the attempted suicides and committed suicides [13, 15, 16], while low education and unemployed young adult men and women had significantly higher rates of attempts [17].

For understanding the growth of the value of rates for men, it is of vital importance to bear in mind wider historical context and political changes in these areas, such as wars, the last one in the region in the 1990s, disintegration of Yugoslavia, NATO air-raids in 1999, political processes leading to the independence of Montenegro in 2006, the transition period and the World Economic Crisis in 2008. Education and the role of men who are expected to be strong and not to show emotions can have adverse effects in this social context. On the other hand, stigma and inadequate psychiatric services represent some of the reasons for not looking always for professional assistance.

## Limitation of the study

We believe that the main limitation of the paper is that it only reflects the situation in the capital of Podgorica where one-third of the population of Montenegro lives. Among them, there are many who migrated from the North and the South of the country, so these data can also refer to them. However, one should bear in mind specific characteristics of the North and the South, cultural and other differences, as well as the factors such as isolation. Out of these reasons, we have started preparations to look into attempted suicides in other parts of the country.

## Conclusions

This is the first study that has examined the attempted suicide rate in Podgorica. The results suggest that women attempt suicides most frequently and that in the observed period there was a decline of attempted suicides in females compared to males (1.93/1.29). Attempted suicide rates ranged from 103 per 100,000 residents in 2016 to 142 per 100,000 residents in 2015, and they belong to lower rates compared to the WHO European multicentre study on parasuicide.

Crude attempt rate was the highest in women in 2012—102.42/100,000 residents—and in men in 2014 and 2015—84.48 and 83.06, respectively, per 100,000 residents.

Poisoning with psychotropic drugs was a dominant method of attempts. The largest number of attempts was in late spring and summer (May, June and July). Unemployment influences men to attempt suicide much more frequently.

## Abbreviations

EW: Emergency Ward of the Clinical Centre of Montenegro
CCM: Clinical Centre of Montenegro
WHO: World Health Organization
MONSTAT: Statistical Office of Montenegro
SPSS: Statistical Package for Social Sciences
SPSS Inc: statistical software
